# Role of Decompressive Craniectomy in Ischemic Stroke

**DOI:** 10.3389/fneur.2018.01119

**Published:** 2019-01-09

**Authors:** Lars-Peder Pallesen, Kristian Barlinn, Volker Puetz

**Affiliations:** Department of Neurology, Carl Gustav Carus University Hospital, Technische Universität Dresden, Dresden, Germany

**Keywords:** stroke, craniectomy, middle cerebral artery infarction, posterior circulation stroke, prognosis

## Abstract

Ischemic stroke is one of the leading causes for death and disability worldwide. In patients with large space-occupying infarction, the subsequent edema complicated by transtentorial herniation poses a lethal threat. Especially in patients with malignant middle cerebral artery infarction, brain swelling secondary to the vessel occlusion is associated with high mortality. By decompressive craniectomy, a significant proportion of the skull is surgically removed, allowing the ischemic tissue to shift through the surgical defect rather than to the unaffected regions of the brain, thus avoiding secondary damage due to increased intracranial pressure. Several studies have shown that decompressive craniectomy reduces the mortality rate in patients with malignant cerebral artery infarction. However, this is done for the cost of a higher proportion of patients who survive with severe disability. In this review, we will describe the clinical and radiological features of malignant middle cerebral artery infarction and the role of decompressive craniectomy and additional therapies in this condition. We will also discuss large cerebellar stroke and the possibilities of suboccipital craniectomy.

## Introduction

The increasing burden of stroke is one of the main challenges for health providers worldwide. Stroke is ranked as the second most common cause of death globally and the most common cause of acquired disability in adults (1, 2). Considerable efforts have been made to enhance the quality of care and medical management in ischemic stroke patients. Intravenous thrombolysis with rt-PA administered within 4.5 h from symptom onset can significantly improve patients' outcome (3). Furthermore, evidence from recent randomized controlled trials underlines the efficacy of endovascular treatment with mechanical removal of occluding blood clots via catheterization (3). However, only a minority of patients (up to 25% in well-organized stroke centers) receive intravenous thrombolysis, and its benefit in large vessel occlusion is limited by an overall low recanalization rate of approximately 20% (4). And although endovascular thrombectomy (EVT) has been shown to be effective in large vessel occlusive stroke within 24 h from stroke onset, it is hampered by the availability of EVT-capable centers (5–9). Furthermore, the patients may suffer from significant ischemic brain damage despite timely recanalization, a situation coined by the term “futile recanalization (10).”

**Figure 1:**
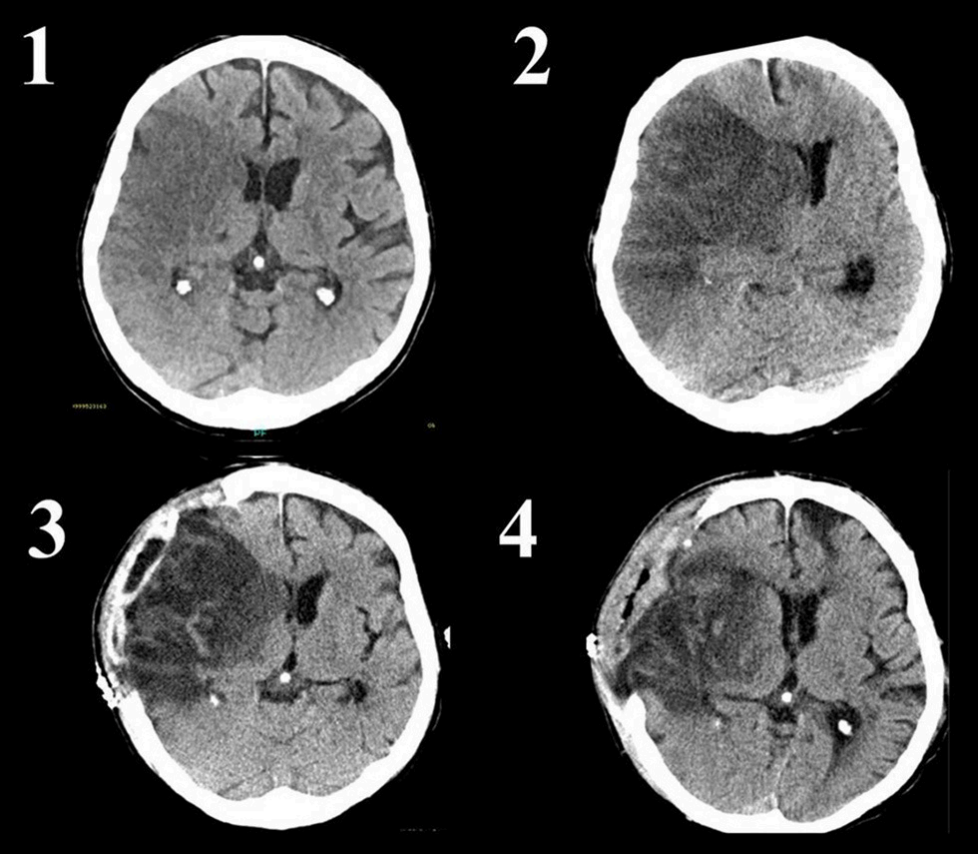
Native CT scans of a patient with infarction of the complete right middle cerebral artery territory. A systemic or endovascular therapy was not conducted due to late arrival and already visible ischemic changes. Image 1 shows the ischemic tissue as darker (hypodense) area without significant mass effect. Image 2 reveals progressive edema of the ischemic tissue with visible midline shift. The occipital horn of the left lateral ventricle is enlarged due to disturbance of cerebrospinal fluid circulation. The patient was alert but deteriorated with reduced level of consciousness immediately prior the CT. Image 3 shows a CT-scan 1 day after decompressive craniectomy. The midline shift and enlargement of the left occipital horn are decreasing, whereas brain tissue is herniating through the skull defect. Image 4 is a CT scan 8 days after decompressive surgery. Midline shift has nearly normalized. The now visible defect on the left frontal part of the brain is caused by an old injury of the patient.

Patients with large hemispheric infarction may suffer from increasing intracranial pressure (ICP) resulting in cerebral herniation and subsequent mechanical and ischemic damage of healthy cerebral territories (11). With decompressive craniectomy (DC), a proportion of the skull is surgically removed to allow the edematous brain tissue to herniate to the outside and thus preventing neuronal damage in other regions of the brain (12) (Figure 1). Two principal groups of stroke patients who may benefit from craniectomy can be distinguished: First, patients with large cerebellar infarction and subsequent suboccipital craniectomy (SOC); and secondly patients with large infarction of the middle cerebral artery territory, also called malignant middle cerebral artery infarction (MMCAI). The latter will be the main topic of this review, therefore we will only briefly comment on surgical options in patients with space-occupying cerebellar infarction.

## Malignant Middle Cerebral Artery Infarction

In 1–10% of all patients with acute middle cerebral artery occlusion, the subsequent ischemic stroke can be classified as “malignant,” defined by ischemic brain tissue large enough to cause considerable increase of ICP and potential cerebral herniation (13). Clinically, the patients present with severe hemispheric symptoms including hemiparesis or hemiplegia, loss of visual field, gaze deviation and, depending on the affected hemisphere, neglect or aphasia. Patients may also show an impaired level of consciousness, nausea, vomiting, papillary changes and papilledema as signs of increased ICP (13). The severity of neurological deficits is usually measured with the National Institute of Health Stroke Scale (NIHSS) score, with higher scores indicating more severe deficits (range 0–42). Patients with MMCAI will typically have scores >15 points if the non-dominant hemisphere is affected and >20 points if the dominant hemisphere is affected (14). The long-term functional outcome of patients with stroke is typically measured with the modified Rankin Scale (mRS) score, with 0 indicating no symptoms and 6 indicating death (Table 1).

**Table 1:** Modified Rankin Scale (mRS).

**Score**	**Description**
0	No symptoms
1	No significant disability. Able to carry out all usual activities, despite some symptoms
2	Slight disability. Able to look after own affairs without assistance, but unable to carry out all previous activities.
3	Moderate disability. Requires some help, but able to walk unassisted.
4	Moderately severe disability. Unable to attend to own bodily needs without assistance, and unable to walk unassisted.
5	Severe disability. Requires constant nursing care and attention, bedridden, incontinent.
6	Dead

Not all patients with middle cerebral artery occlusion develop MMCAI and several studies have attempted to establish predictors of possible mass effect with subsequent clinical deterioration. In a prospectively collected dataset, 19% of patients with ischemic stroke due to middle cerebral artery occlusion had MMCAI with higher NIHSS scores being an independent predictor (15). Furthermore, history of arterial hypertension, increased blood pressure following the first 12 h after stroke onset, female sex, and congestive heart failure have been identified as independent predictors in earlier studies (14, 16). Younger age was also associated with MMCAI, possibly due to lack of age-dependent brain atrophy leading to earlier mass effect of ischemic territories, and fewer intracerebral vessel collaterals due to lower rates of atherosclerotic stenosis in this population (14, 17).

Several neuroradiological predictors for MMCAI have been identified. CT of the brain is still the most widely used imaging method in the assessment of acute stroke (18). Whilst ischemic changes appear as hypodense areas in the affected territories, proximal occlusion of the middle cerebral artery can be detected with CT angiography (19). In cases of middle cerebral artery occlusion, ischemic changes on plain CT in more than 50% of the corresponding territory are independently associated with fatal brain swelling (14, 16). Measurement of optic nerve sheath diameter, eyeball transverse diameter and the ratio of both on plain CT may identify patients with high risk of developing a MMCAI (20). A similar approach has been made with duplexsonography of the optic nerve (21). CT Perfusion may also be useful in the detection of malignant edema in ischemic stroke patients (22–24). Furthermore, clot burden, proximity of clot in the vessel, permeability, and poor intracranial collaterals have been described as CT based predictors for malignant cerebral edema (25).

Imaging assessment of stroke by MRI has the advantage of higher sensitivity for early ischemic changes than CT; however, the examination is more time consuming and suffers from lower availability (18). In cases of middle cerebral artery stroke, one study found a high risk of herniation in patients with an infarct volume >145 cc on diffusion-weighted-images (DWI), whilst another analysis found a high specificity of 98% for the development of MMCAI if the DWI lesion was >82 cc (15, 26).

Prognosis of patients with MMCAI is poor with a mortality rate of approximately 80% if treated conservatively (13, 27). There is only insufficient evidence that additional non-surgical therapeutic regimes other than specialized care on a stroke unit or intensive care unit can improve patients' outcomes. Hence DC should be considered in patients with MMCAI.

## Decompressive Craniectomy in Malignant Middle Cerebral Artery Stroke

### Current Evidence–Randomized Controlled Trials

The procedure of DC to reduce ICP due to cerebral edema is more than 100 years old and numerous studies have addressed this issue so far (28). In the following we will focus on randomized controlled trials (RCTs, Table 2) and meta-analyses. These studies differ in their design, inclusion criteria and primary and secondary endpoints. All studies have in common that patients were randomized either to surgery in form of DC or to best medical care.

**Table 2:** Overview of the randomized controlled trials (RCTs).

**Study name**	**Age (years)**	**Inclusion from symptom onset (hours)**	**Imaging criteria**	**Clinical criteria**	**Primary outcome parameter**	**Main finding**	**Patients included, n (DC/BMT)**
DECIMAL	18–55	<24	>50% ischemic MCA territory; MRI-DWI infarct volume >145 cc	NIHSS >15; NIHSS 1a ≥1	mRS 0–3 at 6 months	52.5% absolute mortality reduction with DC compared to BMT (*p* < 0.0001); no significant difference between DC and BMT regarding mRS 0–3	38 (20/18)
DESTINY I	18–60	>12 to < 36	≥2/3 MCA territory with basal ganglia; with/without ACA/PCA territory	NIHSS >18 (non-dominant) or >20 (dominant); NIHSS 1a ≥1	Sequential design: mortality after 30 days; mRS 0–3 vs. 4–6 at 6 months	Mortality reduction from 88% to 47% with DC after 30 days (*p* = 0.02)	32 (17/15)
HAMLET	18–60	<96	≥2/3 MCA territory; formation of space occupying edema	NIHSS ≥16 (right) or ≥21 (left); NIHSS 1a ≥1; GCS < 13 (right-sided) or GCS (eye and motor score) < 9 (left-sided)	mRS 0–3 vs. 4–6 at 12 months	DC with no effect on primary outcome measure but significant reduction of case fatality (ARR 38%)	64 (32/32)
Zhao et al.	18–80	<48	≥2/3 MCA territory; with/without ACA/PCA territory; space-occupying edema	GCS (eye and motor score) ≤ 9	mRS 0–4 vs. 5–6 at 6 months	Reduction of mortality (DC 12.5% vs. BMT 60.9 %, *p* = 0.001) and mRS 5–6 (DC 33.3% vs. BMT 82.6 %, *p* = 0.001)	47 (24/23)
HeADDFIRST	18–75	<96	≥50% ischemic MCA territory(<5h) or complete MCA infarction (<48h)	NIHSS ≥18; NIHSS 1a <2	survival 21 days	Non-significant reduction of mortality at 21 days (DC 21% vs. BMT 40%, *p* = 0.39)	24 (14/10)
DESTINY II	>60	<48	≥2/3 MCA territory with basal ganglia	NIHSS >14 (non-dominant) or >19 (dominant), reduced level of consouscness on NIHSS	mRS 0–4 at 6 months	Significant reduction of severe disability (mRS scores 5–6: DC 38% vs. BMT 18%, *p* = 0.04)	112 (49/63)
Slezins et al.	>18	<48	≥2/3 MCA; with/without ACA/PCA territory or cerebral infarct volume >145 cc	NIHSS >15	mRS 0–4 vs. 5–6 at 12 months	Significant mortality reduction (DC 45.5% vs. BMT 7.7%, *p* = 0.03)	24 (11/13)
HeMMI	18–65	≤72	≥2/3 MCA territory; with/without ACA/PCA territory	GCS 6–14 (right-side) or 5–9 (left-side); GCS 15 and NIHSS ≥1a	mRS 0–3 vs. 4–6 at 6 months	No significant differences (DC 23.1% vs. BMT 38.4%, *p* = 0.476)	29 (16/13)

*ACA, indicates anterior cerebral artery; ARR, absolute risk reduction; BMT, best medical treatment; DC, decompressive craniectomy; GCS, Glasgow Coma Scale; MCA, middle cerebral artery; mRS, modified Rankin Scale; NIHSS, National Institute of Health Stroke Scale; PCA, posterior cerebral artery*.

- In the multicenter ***DE*compressive** ***C*raniectomy** ***I*n** ***MAL*ignant MCA Infarction (DECIMAL)** trial from France, patients aged 18–55 years with MMCAI were included and either assigned to DC with best medical care or best medical care only (29). MMCAI was defined by three criteria: NIHSS score >15 points (including at least one of three points in the section “reduced consciousness”), involvement of more than 50% of the middle cerebral artery vascular territory on plain CT, and infarct volume of more than 145 cc on MRI- DWI. In almost 4 years, 38 patients were included with 20 patients randomized to DC and 18 to best medical care only. Primary outcome parameter was “favorable functional outcome” 6 month after the index event, defined as mRS scores of 0–3. The safety monitoring committee recommended stopping the study due to slow recruitment, significant difference of mortality in the two groups, and to organize a pooled analysis with the other European RCTs (see below). Looking at the primary outcome parameter, 25% of the patients in the DC group had a favorable functional outcome compared with 5.6% of patients in the best medical treatment group (*p* = 0.18). After 1 year, these numbers increased to 50 vs. 22.2%, respectively (*p* = 0.10). Regarding mortality, DC lead to a 52.8% absolute reduction of death, whereas only 4 out of the 18 patients (22.2%) in the non-surgery group survived.

- In the ***De*compressive** ***S*urgery for the** ***T*reatment of Malignant** ***In*farction of the Middle Cerebral Arter*y* (DESTINY)** trial conducted in Germany, patients aged 18–60 years were randomized either to surgery or conservative therapy (30). Due to the expected overwhelming advantage of DC regarding mortality, this study used a sequential design for outcome parameters: first endpoint was mortality at 30 days. After this endpoint was reached, enrollment was interrupted and the sample size was recalculated based on good functional outcome, defined as mRS scores of 0–3 vs. 4–6, at 6 months. Clinical inclusion criteria were a NIHSS score of >18 if the non-dominant hemisphere was affected and >20 if the dominant hemisphere was affected. Furthermore, patients had to have a decreased level of consciousness of ≥1 on the item 1a of the NIHSS score. Imaging criteria were affection of >2/3 of the middle cerebral artery territory including at least a part of the basal ganglia. Patients could be included if the ipsilateral anterior or posterior cerebral artery territory were also infarcted. After the inclusion of 32 patients, a significant reduction in mortality was evident with survival of 15 of 17 (88%) patients in the DC group compared with 7 of 15 (47%) patients in the conservative group. After 6 and 12 months, there was no statistically significant difference in the rate of favorable functional outcome in both patient groups (47 vs. 27%, respectively; *p* = 0.23). The projected sample size was calculated as 188 patients, but the steering committee recommended the termination of the study in favor of a pooled analysis of the three European RCTs (see below).

- The Dutch ***H*emicraniectomy** ***A*fter** ***M*iddle Cerebral Artery Infarction With** ***L*ife-Threatening** ***E*dema** ***T*rial (HAMLET)** included patients 18–60 years old (31). Patients could be randomized up to 96 h from symptom onset, imaging criteria for inclusion was affection of two-third or more of the middle cerebral artery territory with formation of space-occupying edema. Regarding clinical status prior randomization, patients had to have a NIHSS score of ≥16 for right-sided ischemia and of ≥21 for left sided ischemia as well as a decreased level of consciousness defined by a Glascow Coma Scale ≤ 13 for right-sided lesions or an eye and motor score of ≤ 9 for left-sided lesions. The primary endpoint was defined as “good outcome” (mRS scores 0–3 vs. 4–6) at 12 months. Secondary outcome measures included mRS score at 3 years (32). Of 64 patients included, 50% were either randomized to DC or best medical care. After 1 year, DC had no effect on good functional outcome (25 vs. 25%, respectively; absolute risk reduction [ARR] 0%, 95%CI −21–21; *p* = 1.00), whilst significantly reducing case fatality (ARR 38%; 95%CI 15–60; *p* = 0.002). In the three-year analysis, DC also had no effect on good functional outcome (26 vs. 25%, ARR 1%, 95%CI −21–22; *p* = 0.94). The study was stopped prematurely for futility as it was considered highly unlikely that a significant difference of the primary outcome parameter between the two groups could be detected.

- A **Chinese study**, conducted at four study sites, included patients aged 18–80 years (33). This study was stopped after 47 patients were enrolled due to a significant difference in poor outcome (defined as mRS score 5–6) favoring DC. After 6 months, 8 of 24 (33.3%) patients who received DC compared to 19 of 23 patients (82.6%) in the conservative arm had a mRS score >4 [adjusted risk ratio [aRR] 49.3%, 95%CI 24.9–73.7; *p* = 0.001], and 14 of 23 (60.9%) patients who received best medical care vs. 3 of 24 (12.5%) patients who received DC had died (aRR 48.4%, 95%CI 24.4–72.3; *p* = 0.001). There was no significant difference between both groups regarding poor functional outcome (mRS scores >3) at 6 months and 12 months (*p* = 0.209 and *p* = 0.272, respectively).

- The ***He*micraniectomy** ***a*nd** ***D*urotomy Upon** ***D*eterioration** ***F*rom** ***I*nfarction-*R*elated** ***S*welling** ***T*rial (HeADDFIRST)** was a randomized pilot study to gain information for the design of a phase III trial (34). Based in the United States, a two-step approach for the inclusion of patients was selected: first, all patients aged 18–75 years with unilateral middle cerebral artery infarction who were responsive to minor stimulation and had a NIHSS score ≥ 18 points were screened. If these patients fulfilled the imaging criteria (more than 50% of the middle cerebral artery territory affected on CT performed <5 h from symptom onset or complete infarction on CT performed <48 h from symptom onset), patients were eligible for enrollment and treated according to standardized medical management, closely monitored for clinical deterioration. Patients were eligible for randomization to best medical care vs. DC if at least one of the following criteria were met: midline-shift of the horizontal anterior septum pellucidum of ≥7.5 mm with unchanged or worsened neurological status, or midline-shift of the horizontal pineal ≥4 mm with depression of arousability to the level of effortful awakening. After the screening of 4,909 patients, only 26 patients were randomized of whom 10 patients received best medical treatment and 14 patients received additional DC (one patient was not treated according to protocol due to the decision of the treating physician and from another patient the spouse withdrew consent). After 21 days, 4 of 10 (40%) patients in the conservative arm compared with 3 of 14 (21%) patients who received DC had died (primary study endpoint, *p* = 0.39). At 6 months, the mortality remained 40% (4 of 10 patients) in the best medical treatment group compared with 36% (5/14) in the DC arm.

- After the effect of DC on improved functional outcome had been demonstrated in the pooled analyses of the European randomized controlled trials in patients aged <60 years (see below), the **DESTINY II** trial sought to analyze the effect of DC in patients >60 years old (35). The primary endpoint was a mRS score from 0 to 4 at 6 months. Besides age, patients had to have a NIHSS score >14 (or 19, if the non-dominant hemisphere was affected), a reduced level of consciousness and imaging evidence of infarction in at least two thirds of the middle cerebral artery territory. In 13 German centers a total of 112 patients were enrolled. The data and safety monitoring board recommended to stop enrollment after 82 patients had been assessed clinically at 6 months. Median age was 70 years in both groups. Regarding the primary end point in the intention-to-treat population, 20 of 49 patients (41%) in the DC group vs.10 of 63 patients (16%) in the conservative group had a mRS score of 0–4 (bias corrected 38 vs. 18%, [Odds ratio]OR 2.91, 95%CI 1.06 to 7.49; *p* = 0.04). Mortality at 12-months was 43% (20/47) of the patients who received DC vs. 76% (47/62) in the conservative arm. No patient, neither in the control group nor the DC group, had a mRS score of 0–2 (i.e., functional independence), and only 7% of the patients who underwent DC and 3% of patients in the conservative group had a mRS score of 3 at 12 months (i.e., able to walk without assistance).

Further monocentric studies have assessed the effect of hemicraniectomy on functional outcome in randomized trials.

- One **monocentric study from Latvia** enrolled 28 patients, inclusion criteria were age 18 years, MMCAI defined by CT or MRI with at least 50% infarction in the middle cerebral artery territory (or 145 cc infarct volume), NIHSS score >15 points and possibility to perform surgery within 48 h after symptom onset (36). Primary endpoint was mRS score 0 to 4 vs. 5–6 at 1 year. After exclusion of 3 patients due to time frame violation (surgery >48 h) and one patient due to absence of increased ICP after implantation of a monitoring gauge, 24 patients were finally analyzed of whom eleven (45.8%) received DC and 13 (54.2%) patients best medical treatment. After 1 year, 5 of 11 patients (45.5%) with DC survived compared to 1 of 13 patients (7.7%) in the best medical treatment group (p = 0.03). Among the survivors, 3 of 5 patients in the DC group had a mRS score of 3 and two patients had a mRS score of 2, whereas the surviving patient with best medical management had a mRS score of 4.

- The second monocentric study **(*He*micraniectomy for** ***M*alignant** ***M*iddle cerebral** ***I*nfarction, HeMMI**) was conducted in the Philippines and included patients aged 18–65 with middle cerebral artery infarction, a Glagow Coma Scale (GCS) score of 6–14 for patients with right sided infarction or 5–9 for patients with left sided infarction, or a GCS score of 15 but clinical deterioration of ≥1 point in the consciousness item of the NIHSS score, and infarction of more than 50% of the middle cerebral artery vascular territory on plain CT (37). Primary outcome parameter was mRS score 0–3 vs. 4–6. Secondary outcome parameters were mRS scores 0–4 vs. 5–6 and mortality. Of 29 patients enrolled, 16 (55.2%) received DC and 13 (44.8%) received best medical care. The study is in so far unique, as three patients in the conservative arm eventually received DC due to secondary deterioration and one patient in the DC group did not receive surgery due to acute myocardial infarction. Three patients in the DC arm and two patients in the conservative arm were lost to follow-up. Finally 24 patients (13 [54.2%] with DC vs. 11 [45.3%] with best medical care) were analyzed. At 6 months, there was no statistically significant difference between the two groups regarding all primary and secondary outcome parameters.

- Another monocentric trial, ***DE*compressive surgery for the treatment of** ***M*alignant** ***I*nfarction of the middle cerebral artery: a randomized, controlled trial in a** ***TUR*kish population (DEMITUR)** was conducted in Turkey. To the best of our knowledge, this study has not yet been published.

### Current Evidence–Meta-Analyses

As mentioned above, the first meta-analysis was conducted with pooled data of the first three European RCTs (HAMLET, DECIMAL and DESTINY) (38). The design of this analysis was developed when the studies themselves were still recruiting patients and the outcome measures were defined without knowledge of the results of the individual studies. Patients aged 18–60 were included and primary endpoint was a dichotomized mRS score at 1 year (0–4 vs. 5–6). Secondary endpoints were case fatality at 1 year and a dichotomized mRS score of 0–3 vs. 4–6. Only patients with surgery performed within 48 h after symptom onset were included. In summary, the data of 93 patients were analyzed with 51 patients (55%) randomized to DC and 42 patients (45%) to conservative treatment. Regarding the primary outcome measure, significantly more patients in the best medical treatment group had a mRS score of 5–6 (32/42 [76.2%] vs. 13/51 [25.5%], OR 0.10, 95%CI 33.9–68.5; *p* < 0.0001). The difference between the two groups remained significant on the outcome parameter mRS score 4–6 (conservative arm 33/42 [78.6%] vs. DC arm 29/51 [56.9%]; OR 0.33, 95%CI 4.6–40.9), *p* = 0.014) and death (30/42 [71.4%] vs. 11/51 [21.6%]; OR 0.10, 95%CI 33.3–67.4, *p* = 0.0001) at 12 months.

- A Cochrane review of the three European trials included 134 patients aged 60 or younger (69 patients [51.5%] randomized to DC and 65 patients [48.5%] randomized to best medical treatment) (39). DC significantly reduced the risk of death (OR 0.19, 95%CI 0.09–0.37) and very poor functional outcome (mRS scores 5 or 6; OR 0.26, 95%CI 0.13–0.51) at the end of follow-up period, whilst there was no significant difference regarding mRS scores 4 to 6 (OR 0.56; 95%CI 0.27 to 1.15).

- One meta-analysis published in 2015 included the four European trials (HAMLET, DECIMAL, DESTINY I and II) as well as HeADDFIRST and the Chinese multicentric study, comprising a total of 317 patients (156 [49.2%] in the surgery arm and 161 [50.8%] in the conservative arm) (40). Individual patients were analyzed and a pooled odds ratio was calculated. Here a significant reduction of mortality 6 months after the index event emerged (OR 0.19, 95%CI 0.10–0.37). This difference remained significant at 12 months (OR 0.17, 95%CI, 0.10–0.29). Patients with DC more often achieved a mRS score of 4 at six (OR 3.29, 95%CI 1.76–6.13) and 12 months (OR 4.43, 95%CI 2.27 to 8.66). A similar Meta-Analysis including the same trials came to comparable results (41).

- The most recent Meta-Analysis was published in 2016 and included DECIMAL, HAMLET, DESTINY I, and II, HeADDFIRST, the Chinese multicentric study and the monocentric study from Latvia (42). In summary, 338 patients were included in this analysis with 165 (48.8%) allocated to DC and 173 (51.2%) to best medical care. Regarding death, the authors found that the patients who received DC had a significantly lower mortality (RR 2.05, 95%CI 1.54–2.72; *p* < 0.00001). Surgery increased the likelihood to survive with a mRS 0 to 3 (RR 1.58, 95%CI 1.02–2.46; *p* = 0.04) or mRS 0 to 4 (RR 2.25, 95% CI 1.51–3.35, *p* < 0.0001).

In summary, these studies show a striking advantage of the surgical therapy concerning mortality. This seems to be achieved at the expense of a greater share of patients surviving with a mRS score of 4 and higher. It should be noted that most stroke studies have defined favorable functional outcome as mRS scores 0 to 3, 0 to 2 or even 0 to 1 (in a general stroke population), whereas some MMCAI trials and above mentioned meta-analyses adopted a definition of mRS scores 0–4 for favorable functional outcome instead. This may be justified by the fact that – due to the severity of the disease - it is unlikely that a decent amount of patients with MMCAI could survive with the ability to walk without assistance. However, the definition of “favorable outcome” in these patients remains conflicting (43). Although DC and its technique may be comparable in all studies, best medical treatment was only defined in some of the trials, and we can assume that patients in both treatment groups were treated differently and not all of these differences were reported. The DESTINY II trial showed that DC is also effective in patients aged older 60 years, therefore a strict age threshold for the selection of patients who may qualify for surgical therapy cannot be recommended (35). It should be noted that the percentage of patients with severe disability was significantly higher (19 vs. 4%) and the percentage of patients with moderate disability significantly lower (6 vs. 43%) when compared with patients ≤ 60 years.

### Timing for DC

Although all RCTs defined a time window of inclusion, none of the aforementioned trials addressed the issue of the ideal timing for DC. The DECIMAL trial demanded randomization not later than 24 h after symptom onset with start of surgical procedure no later than 6 h after randomization (29). In DESTINY I, patients could be randomized if a surgical procedure could be performed between 12 and 36 h after symptom onset, with surgery performed not later than 6 h after randomization (30). In contrast, the HAMLET trial allowed patients to be randomized up to 96 h after symptom onset, with start of treatment up to 3 h after randomization (31). Here, median time from onset of symptoms to randomization was 41 h in the surgical arm and 45 h in the best medical treatment arm. About one third (34%) of all patients in the surgical arm were randomized later than 48 h after the index event, compared to 44% in the conservative arm. As HAMLET was negative regarding its primary endpoint, there is currently no evidence that DC improves functional outcome when it is delayed for >48 h and up to 96 h after stroke onset. Moreover, in the Latvian monocentric trial, three patients underwent surgery later than 100 h after symptom onset, however, none of these patients survived (36). The European meta-analysis included only patients with surgery performed no later than 48 h after symptom onset, and DESTINY II followed this pattern (29, 35).

Physicians commonly face the dilemma either to wait until patients with large hemispheric stroke deteriorate clinically, thus accepting a risk of secondary tissue damage due to increased ICP before DC is initiated—or to perform DC preemptive before clinical deterioration, accepting to treat patients aggressively who potentially may not require DC and therefore do not benefit from this procedure. Cerebral edema due to ischemic stroke is expected to culminate on day 2–5 after the index event (11, 44). However, although almost 70% of patients with stroke deteriorate due to cerebral edema within 48 h after symptom onset, roughly one third of patients experience worsening after this time frame (45). There are only few publications that address the timing of DC. In a national inpatient sample analysis from the United States, from a total of 1,301 patients with DC after stroke, 287 patients (22.1%) underwent surgery within 24 h, 726 (55.8%) within 48 h, and 999 (76.8%) within 72 h (46). The impact of timing was analyzed continuously and dichotomized according to the aforementioned time windows. Regarding in-hospital mortality, neither the continuously (OR 1.06, 95%CI 0.97–1.15; *p* = 0.21) nor the dichotomously conducted analyses showed a significant difference (OR 1.03, 95%CI 0.74–1.42; *p* = 0.87; OR 1.00, 95%CI 0.76–1.33; *p* = 0.98 and OR 1.11, 95%CI 0.80–1.55; *p* = 0.53, respectively). However, in the continuous analysis, later DC was associated with greater odds of discharge to institutional care (OR 1.17, 95%CI 1.05–1.31; *p* = 0.005) and of sustained poor outcome, defined by the - in this context seldom used - Nationwide Inpatient Sample Subarachnoid hemorrhage Outcome Measure (NIS-SOM) (OR 1.12, 95%CI 1.02–1.23; *p* = 0.02). Using a dichotomized approach, whilst surgery within 24 h compared to 48 h was not associated with different outcomes, DC performed within 72 h increased the odds for discharge to institutional care or poor outcome (OR 1.59, 95%CI 1.08–2.34; *p* = 0.02 and OR 1.52, 95%CI 1.07–2.16; *p* = 0.02, respectively). Although this study supported to perform DC within 72 rather than 48 h, a subgroup analysis showed a strong association of herniation with mortality (OR 1.70, 95%CI 1.14–2.56; *p* = 0.009), discharge to institutional care (OR 1.36, 95%CI 1.06–1.75; *p* = 0.02) and sustained poor outcome (OR 1.31, 95%CI 1.01–1.71; *p* = 0.045), indicating the importance to perform DC before the development of critically increased ICP rather than fixed time windows.

A recent monocentric randomized trial divided patients with MMCAI into two groups with DC performed after clear neurological deterioration vs. ultra-early DC within 6 h after presentation (47). Of 46 consecutively admitted patients, 27 patients (59%) were allocated to surgery after clinical or radiological deterioration and 19 patients (41%) to ultra-early surgery. There was a significant reduction of mortality favoring the ultra-early DC group [14 patients (52%) vs. 2 patients (10.5%), *p* < 0.05]. Furthermore, the authors report a statistically significant improvement of functional outcome in the ultra-early DC group. The study shows unique features especially concerning the best medical treatment with maintenance application of mannitol, administration of intravenous phenytoin and use of corticosteroids. Furthermore, imaging and clinical criteria for inclusion in this study are not described in detail. Therefore, the generalizability of this study must be questioned.

As there is currently no evidence that DC improves functional outcomes when it is delayed for >48 h and up to 96 h after stroke onset, patients with MMCAI who are eligible for DC should receive surgery within 48 h from symptom onset (31, 38, 48).

### Special Care and Additional Therapy in Patients With MMCAI and DC

Evidence supporting sole conservative treatment to control brain edema in patients with stroke is lacking (49). However, it can be assumed that at least some patients receive antiedema therapy in addition to DC, and all trial protocols allowed for corresponding adjuvant therapies according to national guidelines in these patients (29–31, 35). However, treatment protocols differed remarkably between the studies regarding extent and timing of treatment initiation and in most cases were left at the discretion of the treating physicians. Moreover, data on the duration of analgosedation following hemicraniectomy is lacking.

Besides common critical care with airway management, positioning of the patient, optimization of blood pressure and volume status, the following three procedures are commonly discussed as treatment options for patients with MMCAI.

### Measurement of Intracranial Pressure (ICP)

Critically increased ICP can be clinically detected by reduced level of consciousness, brain stem symptoms resulting from transtentorial herniation and an overall worsening of the neurological status. Treatment for intracranial mass effect should ideally be initiated before the onset of clinical symptoms, thus preventing further damage of brain tissue (50). Repeated imaging via CT or MRI can reveal signs of increased ICP like midline shift, damage to primarily unaffected territories of the brain or enlargement of the intracranial cavities as a sign of cerebrospinal fluid circulation disturbance (51). However, it is not a real-time (i.e., bed-side) method and it is therefore challenging to determine the frequency in which these neuroradiological tests should be performed—particularly in patients with unchanged clinical status. Furthermore, whilst MRI can be difficult to perform in these often unstable patients, CT is associated with a notable radiation exposure. Hence the implantation of an ICP probe should be considered in patients with DC after MMCAI.

There is still controversy regarding the usefulness of these probes in patients with ischemic stroke. Whilst some earlier studied had promising results indicating a direct association between ICP values and clinical outcome and neuroradiological findings, other studies have revealed that patients could develop serious mass effect and even papillary disturbances while normal ICP values are collected (52, 53). Furthermore, although ICP values between 7 and 15 mmHg are considered normal and it is usually recommended to treat values above 20–22 mmHg, ICP values should always be seen in context with the cerebral perfusion pressure (CPP), the difference of middle artery pressure (MAP) and ICP (CCP = MAP-ICP) (54–56). Here a CPP value >50–60 mmHg should be achieved (55).

In summary, the ICP measurement can be helpful in the treatment of patients with DC after ischemic stroke; however, the interpretation values should be done in the context with clinical and neuroradiological findings. This is even more important as the ICP does not increase linearly but steeply above thresholds >25 mmHg. Therefore the decision to perform DC should not be based solely on ICP values but on clinical signs and current guidelines.

### Osmotherapy

One of the most common non-surgical ways to reduce elevated ICP is osmotherapy which is overall applied by almost 90% of neurocritical care physicians (57). The basic principle of osmotherapy consists of the administration of certain substances which elevate the blood osmolality but are unable to pass the blood-brain barrier (48, 58). Following the osmotic gradient, fluid is extracted from the brain tissue into the blood stream, therefore reducing intracranial mass effect.

Although the physiological and pathophysiological principles of osmotherapy are reasonable, data regarding its effect on ICP and functional outcome are ambiguous: whilst some studies have found that osmotherapy can effectively reduce ICP, others have failed to do so, and the overall effect on patients' outcomes remains uncertain. A recent prospective cohort study with 922 included patients revealed a higher rate of dependency (97.7 vs. 58.5%; *p* < 0.001) and mortality (46.5 vs. 5.6%; *p* < 0.001) in patients who received mannitol (59). However, <10% of the patients in the study population received mannitol and the authors do not comment if any or how many patients received DC.

The general concept of osmotherapy is not without criticism. One may argue that an intact blood-brain barrier, which is critical to establish an osmotic gradient, is absent in injured brain tissue, therefore the administration of osmotic agents may be without beneficial but even detrimental effects (60).

In summary, the pure effect of osmotherapy and its effect on functional outcome is a matter of debate. Randomized controlled trials regarding the effect of osmotherapy on clinical outcomes are lacking, although several guidelines recommend its usage in ischemic stroke patients (48, 60). With mannitol, glycerol and hypertonic saline solution being the most commonly used substances, there is no clear evidence of benefit of any of these osmotherapeutic regimes (48, 49, 55, 60). Osmotherapy should not be implemented solely based on neuroradiological imaging and clinical examinations but on continuous bed-side ICP monitoring. Additionally, the existing data do not support the prophylactic administration of osmotherapy in patients with ischemic stroke without clear signs of brain edema or the administration in fixed intervals.

### Hypothermia

Although the benefit of hypothermia has been shown in patients with recent resuscitation and in children with peripartal hypoxia, its clinical usefulness in ischemic stroke patients is still uncertain (61–64). Given the fact that fever is associated with worse outcome, the maintenance of normothermia is generally recommended in patients with intracranial mass effect due to stroke (65). However, this is not supported by randomized controlled trials (66).

Three of the most common ways to regulate body temperature in the critical care setting are via medication like non-steroidal anti-inflammatory drugs (for example paracetamol) or cold saline infusions, by surface cooling with ice bags or surface cooling systems (for example ArcticSun®) or by endovascular cooling systems (for example Thermoguard XP®) (67). Every cooling method has its distinctive advantages and disadvantes: whilst ice bags or cold saline infusions are easily available and inexpensive, maintaining the target temperature can be difficult. Furthermore, infusion of cold saline is limited in patients with cardiac failure due to possible hypervolemia. Surface cooling systems are easy to apply but can cause skin irritations and even cold burns. Intravenous cooling systems rely on semiautomatic body temperature control with electronic feedback. However, this goes with the risk of catheter infections and thrombosis.

As mentioned above, data on the benefit of hypothermia in stroke patients is scarce. One study, conducted before the RCTs on hemicraniectomy were published, followed the course of 36 patients with MMCAI of who 19 received moderate hypothermia of 33° whilst 17 patients underwent DC (68). The hypothermia group had a significantly higher mortality (12% in the DC group vs. 47% in the hypothermia group, respectively; *p* = 0.02). However, it should be noted that the patients in the hypothermia group did not receive DC. A recent study compared 53 retrospectively analyzed patients with MMCAI who would have fulfilled the inclusion criteria for the pooled analysis of randomized hemicraniectomy trials and were treated with DC and additional hypothermia (33–34°C) with 58 patients who underwent DC from the three European RCTs (DECIMAL, DESTINY, and HAMLET) (69). Hypothermia had no benefit on favorable functional outcome (mRS sores of 0–3) at 12 months (13/53 (25 %) vs. 24/58 (41%), aRR 0.66, 95%CI 0.38–1.13) but was associated with higher mortality (27/53 (51 %) vs. 46/58 (21%), RR 0.62, 95%CI 0.46–0.84 [results were basically unchanged after adjustement]) in this study.

Upcoming RCTs are investigating the benefit of hypothermia: Eurohyp is including patients with a NIHSS of 6 to 18, randomizing to best medical treatment or cooling for 24 h with a target temperature of 34–35°, either by surface or endovascular cooling systems (70). The trial is not directly aimed at patients with DC and is also suffering from slow recruitment with 98 of 1,500 planned patients enrolled until March 2018 (71). The results of the ***DE*compressive surgery** ***P*lus hypo*TH*ermia for** ***S*pace-*O*ccupying** ***S*troke** (**DEPTH-SOS**) trial that has randomized patients with MMCAI to cooling to 33°C ± 1 for 72 h in addition to DC are expected to be presented in November 2018 (72).

In view of the present evidence, hypothermia cannot be recommended in patients with MMCAI outside of clinical trials.

### Summary of Special Care and Additional Therapy

Given the available evidence, apart from common critical care no definitive recommendation for additional therapies in patients with MMCAI who underwent DC can be given. Treating physicians may utilize certain measures to decrease elevated ICP based on individual decision making. However, one should be aware that none of these therapies are based on RCTs and are associated with possible side effects. An overview of the recommended medical management can be found in Table 3 (73).

**Table 3:** Recommendations for the treatment of patients with Malignat Middle Cerebral Artery Infarction after Decompressice Craniectomy [modified after (73)].

**Clinical parameter**	**Recommendation**
Airway and ventilation	Target pCO_2_: 4.7 – 5.9 kPa; Target pO_2_ > 8kPa; Target SpO_2_ 95–98%
Hemodynamics	Continuous monitoring of ECG and BP Monitor Treat cardiac arrhythmias, Avoid hypotension, tolerate initial transient hypertension Utilize isotonic fluid to maintain euvolemia. Target CPP 50–60 mmHg
Glucose target	Glucose 7.8 – 9.9 mmol/l (avoid hypoglycemia at all times)
Temperature	Maintain normothermia
Miscellaneous	Administer subcutaneous low-molecular-weight heparin for deep venous thrombosis prophylaxis or intermittent pneumatic compression No indication for seizure prophylaxis
Elevated ICP	Elevate head of bed to about 20-30°, keep neck straight to support venous return Start or increase analgesia and sedation Start mechanical ventilation Apply hyperventilation, but only short term Treat seizures, fever, hyperglycemia, respiratory distress, etc. if present Consider osmotherapy Consider barbiturates Consider muscle relaxation

*BP indicates blood pressure; CPP, cerebral perfusion pressure; ECG, electrocardiography; kPa, kilopascal; ICP, intracranial pressure; pCO2, partial pressure of carbon dioxide; SpO2, peripheral oxygenated saturation*.

### Posterior Circulation Stroke

**Figure 2:**
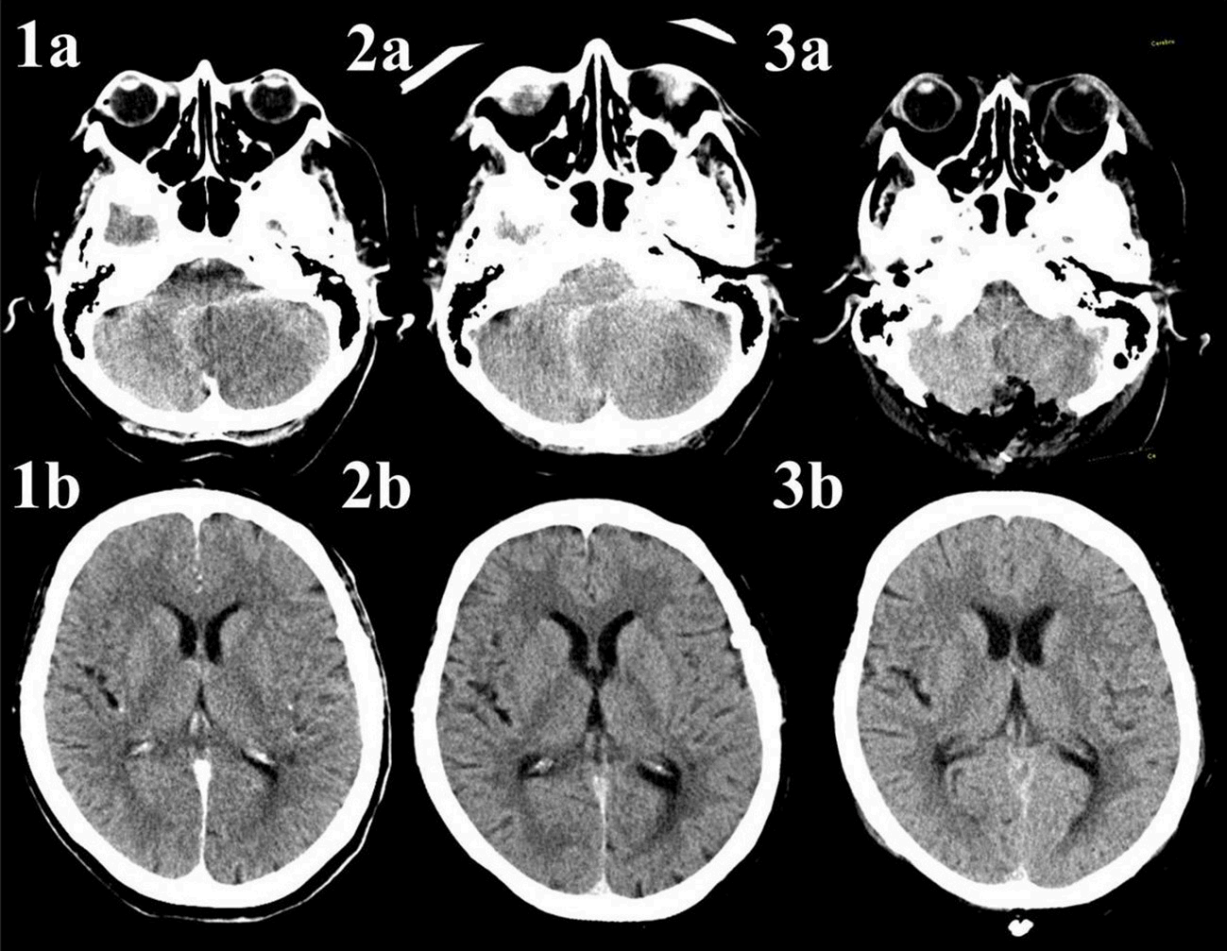
Native CT-scans of a patient with infarction of the left posterior inferior cerebellar artery. The patient underwent no acute treatment due to late arrival and already visible ischemic changes. **Image 1a** shows the hypodense area on the left side of the cerebellum, there are no signs of cerebrospinal fluid circulation disturbance on **Image 1b**. **Image 2a** reveals progressive infratentorial edema with resulting enlargement on the ventricular system due to compression of the fourth ventricle (Figure 2b). **Images 3a**,**b** present a scan 1 day after suboccipital craniectomy. Whilst there is still enlargement of the frontal horns of both lateral ventricles, the third ventricle is slightly smaller, indicating flow restoration. The extraventricular drainage (EVD), implanted during the decompressive craniectomy, is not shown in these images.

About one fifth of all ischemic strokes are located in the posterior circulation and the diagnosis can be challenging due to non-specific symptoms like vertigo, nausea or reduced level of consciousness (74, 75). Large cerebellar infarction with subsequent mass effect followed by transforaminal brainstem herniation and hydrocephalus is the main target of surgical therapy in form of SOC in these patients (76, 77) (Figure 2). Although estimation of prognosis is difficult, patients with cerebellar infarction tend to have a more favorable outcome than patients with other stroke subtypes (78). However, it should be noted that data on long term outcome in these patients is scarce, and that additional ischaemia in adjacent territories like the brainstem and pre-existing conditions may significantly worsen the outcome (79). Large multicenter RCTs are lacking for this situation probably due to the well-known devastating effects of brainstem compression and hydrocephalus. In one of the largest trials, the German-Austrian Space-Occupying Cerebellar Infarction Study (GASCIS), 84 patients with massive cerebellar infarction were prospectively observed, with 34 (40%) receiving surgery, 14 (17%) receiving ventriculostomy and 36 (43%) receiving best medical treatment (80). The only predictor for poor outcome was reduced level of consciousness before treatment (OR 2.8, 95%CI 1.4–5.6). However, patients in GASCIS were not randomized, therefore causing a potential selection bias. Furthermore, 22.2% of patients initially treated with ventriculostomy also received SOC over the course of their hospital stay. Likewise with MMCAI, the timing of surgical therapy is paramount in patients with significant posterior fossa edema due to ischemic stroke (79). Whilst some authors argue that surgical therapy should be considered only when a significant decrease in the level of consciousness is present and that surgery in patients without coma is unproven, others tend to treat more aggressively, as clinical signs or neuroradiological imaging of deterioration may be unspecific or detected too late (48, 81–83). Aggravating this situation is the fact that neuroradiological imaging in the posterior fossa is difficult: although dysfunction of cerebrospinal fluid circulation due to fourth ventricle compression by large cerebellar infarction may easily be spotted on plain CT, early ischemic changes in the posterior circulation can be missed due to bone artifacts (75). Additional test with MRI-DWI, CT angiography source images (CTA-SI) and CT-Perfusion (CTP) may facilitate detection of ischemic changes and estimation of overall outcome (84–86).

Concerning the extent of the surgical procedure, some authors argue that implantation of an extraventricular drainage (EVD) is sufficient, whilst others fear the possibility of upwards herniation across the tentorium (80, 87, 88). Although this question may not be sufficiently answered by current data, we recommend performing SOC with EVD implantation, with the possibility to extract the latter as early as possible if neuroradiological imaging shows sufficient cerebrospinal flow restoration after SOC (79).

In summary, SOC with or without insertion of EVD is an efficient procedure for the treatment of massive posterior fossa edema due to posterior circulation stroke. A strict age-dependent threshold whether to treat aggressively cannot be recommended. The decision to perform surgery should be made depending on pre-existing status of the patients and possible affection of other areas of the brain. As in MMCAI, data on the efficacy of additional therapies is scarce. Similar to MMCAI, we recommend utilizing these individually according to clinical status and neuroradiological imaging.

### Ethical Considerations

Although there is sufficient evidence that DC in patients with MMCAI can be a lifesaving procedure, one should not forget that all patients who survive this condition will suffer from some form of disability. Although 43% of the patients in the pooled analysis of the three European RCTs achieved a mRS score of 0–3, only 7% patients in DESTINY II (i.e., patients older than 60 years) were able to walk without assistance (i.e., mRS score of 3) and no patient regained functional independence (i.e., mRS score of 2) (35, 38). In a recently published retrospective analysis of 66 patients in two tertiary stroke centers, 16% of patients aged 18–75 with DC after MMCI achieved functional independence (89).

The publication of the RCTs lead to an increase of DC in these patients, however, treatment decision making still is challenging as the survival can be at the cost of a life with severe disability, a fate often seen as unacceptable by patients (90, 91). Even if clinical and neuroradiological aspects lead to the recommendation for DC, surgery should only be performed after careful assessment of the patients' attitude toward the possibility of a life without the ability to care of their own bodily needs (43, 91, 92). Making the right decision in patients with MMCAI—whether performing aggressive surgical therapy with an uncertain outcome—represents the difficulty of applying population based study data and experience on individual patients. Further aspects of the ethical conflicts in these patients are discussed in another chapter of this article collection.

## Conclusion

Malignant cerebral infarction is a life threatening condition with a mortality rate of 80% if treated conservatively. Decompressive craniectomy is the only therapeutic approach that is based on data of large randomized controlled trials in this condition. Decompressive craniectomy reduces the mortality rate in these patients, however leaving the majority of patients with at least some disability. Other treatment options like osmotherapy may be used in an individual risk-benefit-assessment, but evidence for these treatments and procedures is scarce. Before the surgical intervention, we recommend careful assessment of the patients' will.

## Author Contributions

L-PP wrote the manuscript. KB and VP were also involved in drafting the manuscript and revising it critically for important intellectual content.

### Conflict of Interest Statement

The authors declare that the research was conducted in the absence of any commercial or financial relationships that could be construed as a potential conflict of interest.
